# Is prophylactic central neck dissection necessary for patients with clinically node-negative papillary thyroid microcarcinoma? A follow-up study of more than 10 years

**DOI:** 10.3389/fendo.2025.1597661

**Published:** 2025-05-16

**Authors:** Zeng-Gui Wu, Wen-Ting Zheng, Li-Jie Chen, Fang-Shuang Zhu, Zhao-Sheng Ma, Fei-Lin Cao, Bin-Bin Cui, Bo-Jian Xie, Xing-Qiang Yan

**Affiliations:** Department of Surgical Oncology, Taizhou Hospital of Zhejiang Province affiliated to Wenzhou Medical University, Linhai, Zhejiang, China

**Keywords:** papillary thyroid microcarcinoma (PTMC), prophylactic central neck dissection (pCND), postoperative complications, clinically node-negative (cN0), total thyroidectomy (TT)

## Abstract

**Background:**

Therapeutic central neck dissection (CND) is strongly recommended for patients with clinically node-positive (cN1) papillary thyroid carcinoma (PTC). However, the role of prophylactic central neck dissection (PCND) remains controversial for clinically node-negative (cN0) PTC, particularly in papillary thyroid microcarcinoma (PTMC). To better elucidate the benefits and disadvantages, we conducted a retrospective analysis with a follow-up of more than 10 years.

**Methods:**

A total of 377 consecutive patients were enrolled in this study between April 2011 and March 2015. 146 patients underwent total thyroidectomy alone (TT group), while 231 patients underwent total thyroidectomy and prophylactic central compartment lymph node dissection (TT+PCND group). Considering the low risk of recurrence, all patients did not receive radioiodine treatment. Post-surgical pathological and preoperative clinical courses, local recurrence, postoperative complications, and follow-up data were all collected.

**Results:**

In the TT+PCND group, 82 patients (35.3%) had occult lymph node metastasis and a higher risk of postoperative complications, including lymphatic leakage, recurrent laryngeal nerve injury, hypoparathyroidism, and accidental parathyroidectomy. Hypoparathyroidism and accidental parathyroidectomy showed a significantly increased risk (p = 0.005, p = 0.049). However, there were no differences in survival and recurrence rates between the two groups.

**Conclusions:**

Routine prophylactic central neck dissection is unnecessary for patients with clinically node-negative papillary thyroid microcarcinoma, as the postoperative complications are significant, while the benefits remain unclear.

## Introduction

Thanks to more accurate imaging technology, numerous reports have demonstrated that the incidence of papillary thyroid carcinoma (PTC) is increasing worldwide (1, 2). Papillary thyroid microcarcinoma (PTMC) is a subset of differentiated thyroid cancers with a favorable prognosis (3), boasting a 10-year survival rate exceeding 97% (4). Lymph node metastasis in the central compartment (CLNM) occurs in 40-60% of patients with PTMC and is a significant risk factor for tumor recurrence in PTC (5, 6). Therefore, therapeutic central compartment neck dissections (CND) are indicated in cases of clinically positive CLNM when preoperative pathology is confirmed (7–9). Micrometastases have been found in the majority of cases and have minimal impact on patient survival (10). Consequently, the necessity of prophylactic central neck dissection (PCND) remains a controversial issue in patients with clinically node-negative (cN0) PTMC. Supporters argue that routine dissection of central lymph nodes is beneficial for accurate postoperative staging and subsequent treatment guidance. As technology advances, the initial operation is conducive to thorough dissection, avoiding the challenges of complete lymph node removal due to postoperative tissue adhesion and scarring in cases of tumor recurrence, which not only affects the thoroughness of radical surgery but also easily leads to complications (11, 12). Conversely, opponents argue that there is no strong evidence to support PCND. Instead, an increasing number of clinical trials have confirmed no clear benefit on locoregional and biochemical recurrence in PCND patients, even leading to more complications (13–16). The 2015 American Thyroid Association (ATA) guidelines recommend that PCND should be considered in patients with T3–4 PTC, while the Chinese guidelines recommend ipsilateral prophylactic central lymph node dissection based on the effective protection of parathyroid glands and nerves (17). To better determine whether PCND is necessary in the surgical treatment of all PTMC, we aim to elucidate the benefits and disadvantages of PCND and the recurrence rate in cN0 PTMC patients in two groups.

## Materials and methods

### Study population and design

**Figure 1:**
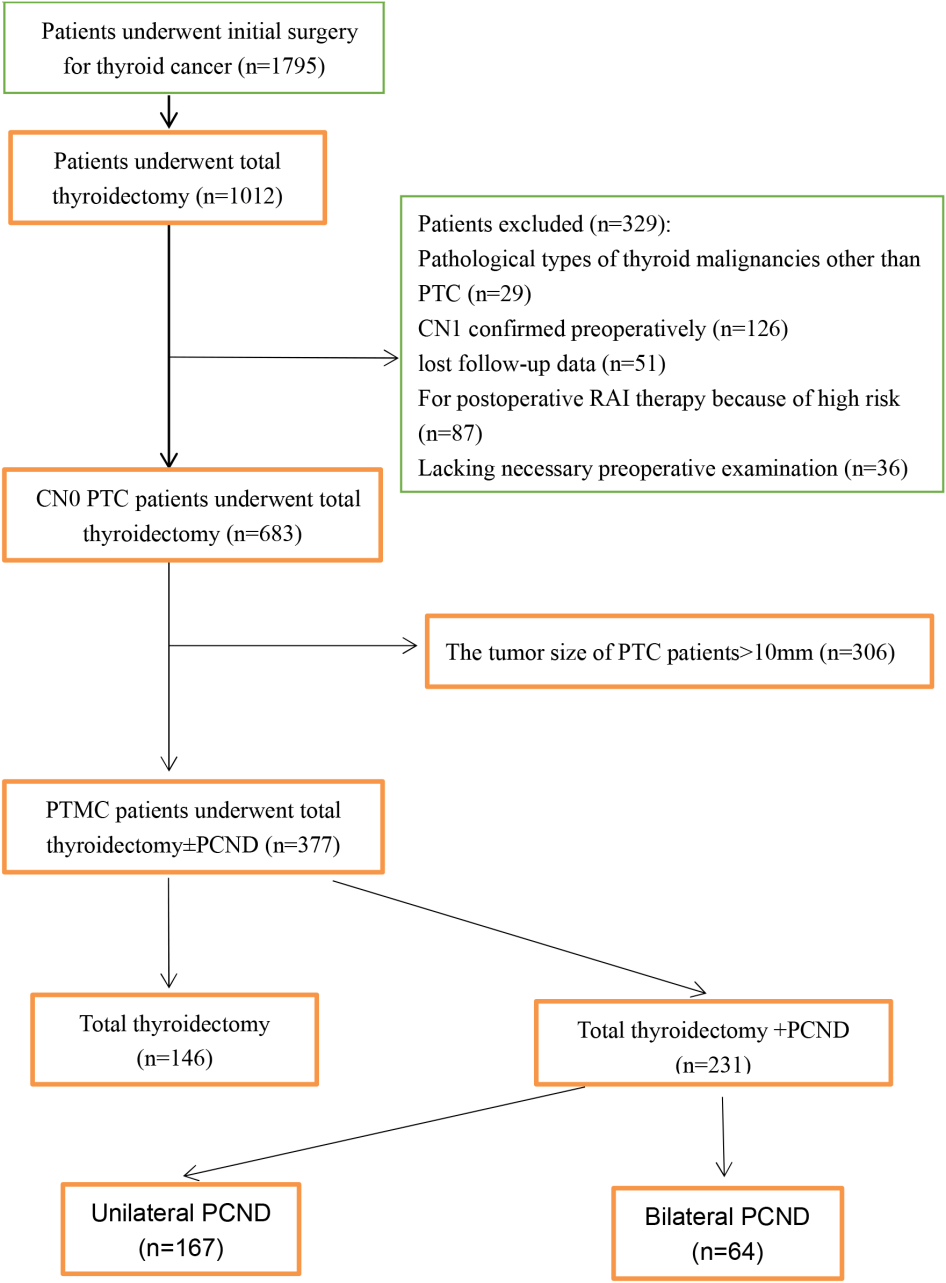
A flowchart showing the inclusion and exclusion criteria and selection steps.

This study retrospectively collected prospective data from the medical record system of the Department of Surgical Oncology at Taizhou Hospital, Wenzhou Medical University. A total of 1795 consecutive patients who underwent initial surgery for thyroid carcinoma between April 2011 and March 2015 were reviewed. Inclusion criteria were as follows (1): primary diagnosis of PTMC (2), cN0 disease (3), total thyroidectomy with or without PCND (4), Complete follow-up data. Exclusion criteria were as follows (1): recurrent thyroid carcinoma (2), lateral cervical lymph node metastasis (3), previous cervical surgery (4), distant metastasis (5), other malignant tumors. Finally, 377 patients were included in this study. The detailed inclusion and exclusion results and steps of selection are shown in the flowchart (**Figure 1**).

### Clinical features and pathological indicators

All patients underwent total thyroidectomy (TT) through traditional open surgery. A routine preoperative cervical ultrasound, interpreted by a senior radiologist, revealed no evidence of lymph node involvement among enrolled patients. PCND was not a routine procedure but was performed based on the surgeon’s assessment of potential risk factors, such as multiple lesions, younger age, larger tumor and Intraoperative conditions, including macroscopically swollen lymph nodes and effectively protection of parathyroid glands and nerves. The central compartment lymph nodes are demarcated by the trachea into left and right regions. Bilateral PCND is performed only when bilateral PTMC is confirmed by preoperative fine -needle aspiration (FNA) or intraoperative pathological evaluation. Otherwise, unilateral PCND is performed. The scope of PCND: up to the lower edge of the cricoid cartilage, down to the suprasternal fossa, both sides to the medial side of the common carotid artery, and back to the tracheoesophageal groove. Complete removal of the anterior laryngeal, anterior tracheal and bilateral parastheal lymph nodes. To minimize nerve injury, intraoperative neuromonitoring (e.g., Medtronic NIM system) is routinely utilized during surgery. The patients with lateral cervical lymph node metastases or residual remnants due to tumor invasion of tissues like the esophagus and trachea, leading to incomplete excision, were excluded, as they required postoperative radioactive iodine 131. At last patients were divided into the PCND (+) group (underwent TT +PCND) and the PCND (−) group (underwent TT alone). Staging was based on pathological data and age, with patients classified as stages I or II. The preoperative and postoperative clinicopathological factors for each group including: age, gender, tumor size, central lymph node metastases, capsular invasion, Hashimoto’s thyroiditis (TgAb or TPOAb level), multifocality, TNM classification, postoperative complication, local recurrence rates, follow-up time, rate of accidental parathyroidectomy and the number of lymph nodes dissected. Routine preoperative laryngoscopy was performed in all patients before and within one month after surgery to assess the function of the vocal cords. Postoperative complications consist of hypoparathyroidism (parathyroid hormone below 10mg/dL), recurrent laryngeal nerve (RLN) injury, lymphatic leakage and accidental parathyroidectomy, of which hypoparathyroidism and RLN injury were divided into transient (<6 months) and permanent (>6 months) according to the duration. Permanent RLN injury was defined as hypomobile or fixed vocal cord movement persisting 6 months after surgery. We routinely monitored serum calcium and parathyroid hormone levels on the 2nd day after surgery and one week after surgery, Persistent low parathyroid hormone levels after 6 months of calcium supplementation were defined as permanent. Lymphatic leakage was defined by an increase in postoperative neck drainage volume and a change in drainage fluid color from bloody to serous, worsening after eating. Inadvertent shearing of the parathyroid glands during surgery is defined as accidental parathyroidectomy.

### Postoperative examination evaluation and follow-up

All patients were reexamined thyroid function one month after operation to adjust the dose of Levothyroxine tablets, then every three months for the first year, and every six months thereafter. The review content including physical examination, full thyroid function, thyroglobulin, thyroid Doppler ultrasound, thyroid CT and so on. None of the enrolled patients underwent 131I therapy due to the absence of a clear indication according to ATA Guidelines. We defined disease-free status as undetectable thyroglobulin levels below 1 ng/mL, undetectable TgAb, and no suspicious lesions on B-ultrasound during follow-up.

### Statistical analysis

we used SPSS version 26 software for statistical analyses to identify differences between groups for specific variables. Quantitative results are reported with mean and standard deviation in the case of a normal distribution. Continuous variables were analyzed by using independent samples t-tests or Mann-Whitney U test. Categorical variables were evaluated using the χ2 or Fisher exact test. P values less than 0.05 were considered statistically significant.

## Results

### Preoperatively basic conditions and pathological factors

The final 377 patients enrolled in our study were divided into two groups.TT group contains 146 patients who underwent total thyroidectomy only, and 231 patients who underwent total thyroidectomy with PCND were classified as TT+PCND group. All surgeries are performed by thyroid specialist with more than 15 years of surgical experience. All basic preoperative conditions and pathological factors of the two groups are described in **Table 1**. The proportion of males was higher in the TT+PCND group compared to the TT group(12.9%vs5.5%), with a statistically significant difference(p=0.018).The median age in the TT group was 49.58 ± 7.87(31-73)years old, which was significantly higher than that in the TT+PCND group, which was 46.93 ± 8.82(26-73)years old(p=0.003). The age of 55 is the most important indicator in the latest version of thyroid cancer TNM staging (18). When patients were divided into those under 55 and those 55 and older, no significant difference was found between the two groups (p=0.165). Thyroid cancer TNM staging was also similar between the two groups(p=0.918). The number of patients with tumor size > 5 mm was significantly more in the TT+PCND group compared to the TT group (70.6% vs 42.5%, p<0.001). In addition, capsular invasion and multifocality were relatively more common in the TT+PCND group compared to the TT group (27.7% vs 16.4%, p=0.012; 25.1% vs 13.7%, p=0.008). Hashimoto’s thyroiditis is relatively common in thyroid cancer, and in our study, it was found that concomitant Hashimoto’s thyroiditis was more prone to lymph node dissection, in 45.9% (n =106) of patients in TT+PCND group and in 16.4% (n =24) of patients in TT group. This difference was found to be significant on statistical analysis (p <0.001).

**Table 1:** Basic preoperative conditions and pathological factors between the TT group and the TT+PCND group.

Clinical factors	TT group (146)	TT+PCND (231)	P
Gender			
Male	8(5.5%)	30(12.9%)	0.018
Female	138(94.5%)	201(87.1%)	
Age (years)	49.5 ± 7.87 (31-73)	46.93 ± 8.82 (26-73)	0.003
Age cohort			0.165
<55 years	108(74.0%)	185(80.3%)	
≥55 years	38(26.0%)	46(19.7%)	
Tumor size			0.000
>5mm,≤10mm	62 (42.5%)	163 (70.6%)	
≤5mm	84 (57.5%)	68 (29.4%)	
CLNM			
YES	0	82 (35.5%)	
NO	146	149(64.5%)	
Capsular invasion			0.012
YES	24 (16.4%)	64(27.7%)	
NO	122(83.6%)	167(72.3%)	
Hashimoto’s thyroiditis			0.000
YES	38(26.1%)	106(45.9%)	
NO	108(73.9%)	125(54.1%)	
Multifocality			0.008
YES	20(13.7%)	58(25.1%)	
NO	126(86.3%)	173(74.9%)	
TNM classification			0.918
I	140 (95.9%)	221 (95.7%)	
II	6 (4.1%)	10 (4.3%)	

TT, total thyroidectomy; TT+PCND, total thyroidectomy + prophylactic central compartment lymph node dissection; CLNM, Lymph node metastasis in the central compartment.

### Postoperative complications

All patients were followed up for at least 10 years, and there was no significant difference in follow-up time between the two groups (134.2 vs 135.7, p=0.448). Regarding postoperative complications between the two groups, all complications were relatively more in TT+PCND group, the specific data are described in detail in **Table 2**. Moreover, postoperative hypoparathyroidism and accidental parathyroidectomy had a statistically significant difference between the two groups (38.5% vs24.9%, p=0.005;6.49% vs2.05%, p=0.049). There were 36 patients (24.9%) with hypoparathyroidism in the TT group, and 35 (23.7%) were temporary. However, in the TT+PCND group, 89 (38.5%) patients had hypoparathyroidism and 5(2.16%) were permanent. Accidental parathyroidectomy in the TT+PCND group included 15 patients, significantly more than the 3 patients in the TT group. There were 4 patients (2.74%) with postoperative recurrent laryngeal nerve injury in the TT group, of which 3 (2.06%) were temporary, lower than 8 patients (3.46%) in TT+PCND group, of which 7 (3.03%) were temporary and one (0.43%) had permanent damage, but the difference was not found to be significantly on statistical analysis (p=0.929). Among other complications, the incidence of lymphatic leakage (1.34% vs 3.46%, p=0.366) did not noticeably differ between the two groups. Among all patients with PCND, occult lymph node metastasis was noted in 35.5% (82/231) of cases. The presence of central lymph node metastasis in these patients was significantly associated with adverse clinicopathological risk factors, such as younger age (p = 0.048), multifocality (p = 0.002). There were no differences in other clinicopathological factors and recurrence rates between the two groups, and the details can be found in **Table 3**. When further subdividing the TT+PCND group of patients into unilateral and bilateral dissection groups, we found that the bilateral dissection group had a higher average number of harvested lymph nodes (6.75vs9.34,p <0.001) and metastatic lymph nodes (0.5vs1.16,p <0.001), and the rate of central lymph node metastasis was also higher in the bilateral PCND group (50.0% vs 29.9%, p=0.004), but there was no significant difference in complications between the two groups except for accidental parathyroidectomy (12.5%vs4.19%,p=0.046), as detailed in **Table 4**.

**Table 2:** Postoperative complications and recurrence between the TT group and TT+PCND group.

Postoperative factors	TT group (146)	TT+PCND (231)	P
Follow-up (months)	134.2 (112-159)	135.7 (111-160)	0.448
Hypoparathyroidism	36 (24.9%)	89 (38.5%)	0.005
Temporary	35 (23.7%)	84 (36.4%)	0.012
Permanent	1 (0.68%)	5 (2.16%)	0.487
RLN injury	4 (2.74%)	8 (3.46%)	0.929
Temporary	3 (2.06%)	7 (3.03%)	0.806
Permanent	1 (0.68%)	1 (0.43%)	0.746
Lymphatic leakage	2 (1.34%)	8 (3.46%)	0.366
Accidental parathyroidectomy	3 (2.05%)	15 (6.49%)	0.049
Recurrence	2 (1.36%)	5 (2.16%)	0.869
Local recurrence	1 (0.68%)	3 (1.29%)	0.960
Thyroglobulin (TG) level>1.0 ng/mL	1 (0.68%)	2 (1.72%)	0.846

TT, total thyroidectomy; TT+PCND, total thyroidectomy + prophylactic central compartment lymph node dissection; CLNM, Lymph node metastasis in the central compartment; RLN, recurrent laryngeal nerve.

**Table 3:** Clinical-pathological features between the CLNM (+) and CLNM (-) in the TT+PCND group.

Clinical-Pathological Features	CLNM (+) 82	CLNM (-) 149	P
Gender			0.029
Male	16 (19.5%)	14 (9.40%)	
Female	66 (80.5%)	135 (90.6%)	
Age (years)	45.59 ± 9.31	48.58 ± 8.28	0.048
Age cohort			0.029
<55 years	72 (87.8%)	113 (75.8%)	
≥55 years	10 (12.2%)	36 (24.2%)	
Tumor size			0.064
>5mm,≤10mm	64 (78.0%)	99 (66.4%)	
≤5mm	18 (22.0%)	50 (33.6%)	
Capsular invasion			0.694
YES	24 (29.3%)	40 (26.8%)	
NO	58 (70.7%)	109 (73.2%)	
Hashimoto’s thyroiditis			0.120
YES	32 (39.0%)	74 (49.7%)	
NO	50 (61.0%)	75 (50.3%)	
Multifocality			0.002
YES	26 (31.7%)	32 (21.5%)	
NO	56 (68.3%)	117 (78.5%)	
Recurrence	3 (3.66%)	2 (1.34%)	0.493
Local recurrence	2 (2.44%)	1 (0.67%)	0.270
Thyroglobulin (TG) level>1.0 ng/mL	1 (1.22%)	1 (0.67%)	0.674
Harvested LNs (mean ± SD)	7.29 ± 5.09	7.62 ± 3.79	0.575

TT, total thyroidectomy; TT+PCND, total thyroidectomy + prophylactic central compartment lymph node dissection; CLNM, Lymph node metastasis in the central compartment.

**Table 4:** Clinical-pathological features between the unilateral and bilateral in TT+PCND group.

Clinical-Pathological Features	unilateral PCND 167	bilateral PCND 64	P
Hypoparathyroidism	64 (38.3%)	25 (39.1%)	0.918
Temporary	61 (36.5%)	23 (35.9%)	0.934
Permanent	3 (1.80%)	2 (3.20%)	0.908
RLN injury	4 (2.40%)	4 (6.25%)	0.302
Temporary	4 (2.40%)	3 (4.69%)	0.631
Permanent	0	1 (1.56%)	0.618
Lymphatic leakage	4 (2.40%)	4 (6.25%)	0.302
Accidental parathyroidectomy	7 (4.20%)	8 (12.5%)	0.046
Central lymph node metastases	50 (29.9%)	32 (50.0%)	0.004
Harvested LNs (mean ± SD)	6.75 ± 3.78	9.34 ± 5.01	0.000
Metastatic LNs (mean ± SD)	0.50 ± 0.53	1.16 ± 1.40	0.000

TT, total thyroidectomy; TT+PCND, total thyroidectomy + prophylactic central compartment lymph node dissection; CLNM, Lymph node metastasis in the central compartment; RLN, recurrent laryngeal nerve.

### Recurrence rate analysis

During the extended follow-up period, there were no tumor-related deaths and no distant metastases in either group of patients. In addition, among them 4 cases had ipsilateral lateral or central lymph node metastases and underwent lymph node dissection as well as RAI ablation therapy after diagnosis, with local recurrence times ranging from 1 to 6 years post-surgery. Another 3 patients exhibited Tg >1.0 ng/ml, but without radiographic evidence, we decided on follow-up observation. As for survival rates and recurrence rates, there was no statistical difference between the two groups (2.16 vs 1.36 p=0.869).

## Discussion

With improvements in ultrasound technology, thyroid microcarcinoma continues to be discovered, and the incidence of thyroid cancer in the United States has tripled over the past 20 years (19), a similar trend has been observed in China (20, 21). PTMC is considered a relatively less biologically invasive cancer, and is even known as an indolent cancer, although it can be aggressive in some patients (22, 23). One of the most important prognostic indicators for PTMC is the presence of lymph node metastases at the initial presentation (24, 25). Micrometastases have been found in up to 80% of cases, with the central cervical compartment involved in 20%-50% of patients (20, 26). When lymph node metastases are identified preoperatively, current guidelines support therapeutic central lymph node dissection, but preoperative ultrasound and other related auxiliary examinations can only detect less than half of the metastases, leading to an underestimation of lymph node metastasis in the central area (27). PCND can provide more precise postoperative staging for PTMC, potentially changing subsequent treatment options and decreasing local recurrence rates, thus improving mortality (28, 29). Opponents argue that PCND will only increase postoperative complications such as hypoparathyroidism and recurrent laryngeal nerve injury, without impacting the long-term prognosis or reducing the recurrence risk (14–16). The need for central lymph node dissection has been thoroughly explored in numerous studies, most of which enrolled patients with PTC, and there are relatively few data focusing on PTMC. Therefore, there is currently no convincing evidence to support performing PCND in cN0 PTMC. Compared to ATA and European guidelines, Chinese experts are more inclined to perform PCND, emphasizing the importance of protecting nerves and parathyroid glands (30). Several articles have recently been published on whether to perform PCND in PTMC. Yang et al. (31)used three Cox regression models to evaluate the correlation between PCND and locoregional recurrence rate (LRR), finding no significant difference in the 15-year LRR between groups and a small absolute benefit for PTMC. Of all 1584 patients enrolled, 1484 underwent thyroidectomy plus PCND, and only 100 underwent thyroidectomy alone, creating a large disparity in enrollment numbers that limits comparability in postoperative complications. Xie et al. (32) showed that the LRR between the non-dissection group and the dissection group was 7 (2.4%) vs. 2 (1.1%), a non-significant difference (χ2 = 0.126, P = 0.169). Except for the hand and foot numbness rates (P < 0.001), which were considerably greater in the dissection group, there was no difference in the remaining postoperative complications between the two groups, such as hypoparathyroidism and hypocalcemia. Both of the above studies enrolled patients over a period of more than 10 years, with many changes in guidelines, and the results may not be very instructive. In our study, we enrolled patients for 4 consecutive years, ending no later than the 2015 ATA publication. The decision to perform PCND after total thyroidectomy is generally at the surgeon’s discretion, considering various risk factors. All of these factors have been referred to as risk factors for central lymph node metastasis in previous studies (23, 33, 34), and all the clinical-pathological markers can be assessed before and during surgery. Patients with Hashimoto’s thyroiditis often have a large number of paratracheal lymph nodes, which can easily be mistaken for metastatic lymph nodes during surgery, resulting in excessive dissection (35). Intraoperative pathological frozen section examination may be able to better identify such enlarged lymph nodes (36).

In our study, which recruited low-risk patients, there were no deaths or distant metastases, and there were only 2 (1.36%) and 5 (2.16%) cases of recurrence in the two groups, respectively. There was no statistical difference between the two groups, which aligns with previous studies (16, 31, 32, 37). Radioactive iodine therapy has played a positive role in patients with insufficient surgical treatment (38), so most studies involve iodine-131 therapy, even in low-risk patients (39). However, iodine-131 therapy requires an isolation environment (40), and none of our patients had residual cancer post-surgery, so none of them received iodine-131 therapy. Increased complications after surgery are one of the main reasons why many experts do not recommend PCND. Common complications include nerve damage, parathyroid gland injury, lymph leakage, and accidental parathyroid gland removal (41, 42). Postoperative hypoparathyroidism is divided into temporary and permanent types. Once a patient develops permanent symptoms, they need to supplement calcium and vitamin D for a long time. Occasionally, they may experience numbness in their hands and feet, which seriously affects their quality of life (43). In our study, the TT+PCND group had a significantly higher incidence of hypoparathyroidism than the TT group, and the difference was statistically significant (38.5% vs. 24.9%, P = 0.005), consistent with previous studies (13–16). The position and number of parathyroid glands are not fixed, and their appearance closely resembles that of fat and lymph nodes. Therefore, they are often misdiagnosed as lymph nodes and subsequently subjected to lymph node dissection. From our data, we can see that the TT+PCND group had a significantly higher rate of accidental parathyroidectomy (6.49% vs. 2.05%, P = 0.049). Therefore, they should not be easily removed unless there is certainty (44). Previous experts have questioned whether bilateral central lymph node dissection has more complications compared to unilateral dissection. Dobrinja et al. (13) subdivided the PCND group between ipsilateral and bilateral central neck dissection, finding no significant difference in terms of RLN injury or hypoparathyroidism. In contrast, we also divided the PCND group into bilateral and unilateral subgroups and identified that there were more cases of accidental parathyroidectomy in the bilateral cohort. Therefore, bilateral dissection must be performed with greater care to identify parathyroid glands.

We believe that there are many factors to consider when deciding whether to perform PCND for patients with cN0 PTMC. Firstly, sufficient preoperative examination and evaluation should be conducted, including high-resolution color Doppler ultrasound and thyroid enhanced CT (45). Secondly, the patient’s baseline characteristics, such as gender, age, tumor size, multiple lesions, and Hashimoto’s thyroiditis, should be considered, and the qualifications of the surgeon should be strictly standardized, as inexperienced thyroid surgeons have relatively increased complication rates (46). Thirdly, fine separation along the thyroid capsule during the operation should be performed. Due to the proximity of the lower parathyroid glands to the central lymph nodes, they are at risk of being damaged during surgery (47). Parathyroid glands should not be easily removed unless there is certainty. At the same time, it is also necessary to carefully investigate whether there are parathyroid glands that have been mistakenly removed from the excised thyroid gland and the cleared lymph nodes. If discovered, they should be transplanted in a timely manner.

Our research has limitations. Firstly, we are a retrospective study with potential selection bias between the two groups. Secondly, the patients we enrolled had a low risk of recurrence, and the recurrence rates of both groups were not high, making it difficult to make a comparison. Thirdly, we mainly rely on medical records to document complications. Incomplete records will definitely lead to underestimation of complications in some patients. Fourth, Postoperative laryngoscopy within one month may miss transient vocal cord paralysis due to potential early functional recovery, resulting in underestimated temporary paralysis rates. It is necessary to conduct large-scale multicenter longitudinal design studies to better evaluate the role of dissection in PTMC treatment.

## Conclusion

In summary, PCND should not be applied to all PTMC patients with cN0 disease. For most extremely low-risk patients, we can assure them that the risk of recurrence will not increase without PCND, and unnecessary postoperative complications can be avoided. Our long-term follow-up data demonstrate that PCND does not improve clinical outcomes while increasing complication risks, providing compelling evidence against its routine application in low-risk cN0 PTMC.

## Data Availability

The raw data supporting the conclusions of this article will be made available by the authors, without undue reservation.
